# Outcomes of severe systemic rheumatic disease patients requiring extracorporeal membrane oxygenation

**DOI:** 10.1186/s13613-021-00819-3

**Published:** 2021-02-09

**Authors:** Pierre Bay, Guillaume Lebreton, Alexis Mathian, Pierre Demondion, Cyrielle Desnos, Juliette Chommeloux, Guillaume Hékimian, Nicolas Bréchot, Ania Nieszkowska, Matthieu Schmidt, Fleur Cohen-Aubart, Pascal Leprince, Charles-Edouard Luyt, Zahir Amoura, Alain Combes, Marc Pineton de Chambrun

**Affiliations:** 1 Service de Médecine Intensive-Réanimation, Hôpital La Pitié–Salpêtrière, Sorbonne Université, Assistance Publique-Hôpitaux de Paris (APHP), Paris, France; 2 Service de Chirurgie Cardiothoracique, Hôpital La Pitié–Salpêtrière, Institut de Cardiologie, Sorbonne Université, APHP, Paris, France; 3 Service de Médecine Interne 2, Institut E3M, Sorbonne Université, Hôpital La Pitié–Salpêtrière, 47–83, Boulevard de L’Hôpital, 75651 Paris Cedex 13, France; 4 Centre de Référence National Lupus Systémique, Syndrome Des Anticorps Anti-Phospholipides Et Autres Maladies Auto-Immunes Systémiques Rares, Paris, France; 5 Institut de Cardiométabolisme Et Nutrition (ICAN), Sorbonne Université, INSERM, UMRS_1166-ICAN, Paris, France

**Keywords:** Systemic rheumatic disease, Extracorporeal membrane oxygenation, Intensive care unit, Vasculitis, Systemic lupus erythematosus, Connective tissue disease, Acute respiratory distress syndrome, Cardiogenic shock

## Abstract

**Background:**

Systemic rheumatic diseases (SRDs) are a group of inflammatory disorders that can require intensive care unit (ICU) admission because of multiorgan involvement with end-organ failure(s). Critically ill SRD patients requiring extracorporeal membrane oxygenation (ECMO) were studied to gain insight into their characteristics and outcomes.

**Methods:**

This French monocenter, retrospective study included all SRD patients requiring venovenous (VV)- or venoarterial (VA)-ECMO admitted to a 26-bed ECMO-dedicated ICU from January 2006 to February 2020. The primary endpoint was in-hospital mortality.

**Results:**

Ninety patients (male/female ratio: 0.5; mean age at admission: 41.6 ± 15.2 years) admitted to the ICU received VA/VV-ECMO, respectively, for an SRD-related flare (*n* = 69, *n* = 38/31) or infection (*n* = 21, *n* = 10/11). SRD was diagnosed in-ICU for 31 (34.4%) patients. In-ICU and in-hospital mortality rates were 48.9 and 51.1%, respectively. Nine patients were bridged to cardiac (*n* = 5) or lung transplantation (*n* = 4), or left ventricular assist device (*n* = 2). The Cox multivariable model retained the following independent predictors of in-hospital mortality: in-ICU SRD diagnosis, day-0 Simplified Acute Physiology Score (SAPS) II score ≥ 70 and arterial lactate ≥ 7.5 mmol/L for VA-ECMO–treated patients; diagnosis other than vasculitis, day-0 SAPS II score ≥ 70, ventilator-associated pneumonia and arterial lactate ≥ 7.5 mmol/L for VV-ECMO–treated patients.

**Conclusions:**

ECMO support is a relevant rescue technique for critically ill SRD patients, with 49% survival at hospital discharge. Vasculitis was independently associated with favorable outcomes of VV-ECMO–treated patients. Further studies are needed to specify the role of ECMO for SRD patients.

## Introduction

Systemic rheumatic diseases (SRDs) are a group of inflammatory disorders (including connective tissue diseases, rheumatic disorders, vasculitides, sarcoidosis, adult-onset Still’s disease…) involving more than one organ and often requiring immunosuppressant therapy [1]. They share common characteristics: multiorgan involvement responsible for end-organ failures; specific treatments causing immunosuppression and infectious complications; and are rare entities with challenging diagnoses and diagnostic difficulties. Outcomes of SRD patients requiring ICU admission remain unclear, with 16%–33% reported in-ICU mortality [1–4].

Extracorporeal membrane oxygenation (ECMO) is a rescue technique used to temporarily replace the heart and/or lung functions of the most severe patients [5, 6]. It may serve as a bridge-to-recovery or a bridge-to-organ transplantation for patients with treatment-refractory heart and/or lung failure(s).

We undertook this study to determine the outcome and identify in-hospital mortality associated factors of critically ill SRD patients receiving ECMO.

## Methods

### Patients

We retrospectively reviewed the prospectively constituted ECMO database of our 26-bed ICU to identify adult SRD patients who received, between January 2006 and February 2020, venoarterial (VA)-ECMO and/or venovenous (VV)-ECMO for heart and/or lung end-organ failure(s). SRD were identified searching in all medical charts a large number of keywords referring to SRD including: systemic rheumatic disease; connective tissue disease; lupus; systemic sclerosis; scleroderma; antiphospholipid; myositis; inflammatory myopathy; Sharp; Sjögren; Gougerot; rheumatoid arthritis; spondylarthritis; vasculitides; Goodpasture; antineutrophil cytoplasmic antibodies; proteinase 3; myeloperoxidase; Henoch-Schönlein; sarcoidosis; Still’s disease; eosinophilia; myasthenia; neuromyelitis optica… Our tertiary ICU is an ECMO-referral center for Greater Paris. Patients with the following SRDs were considered for inclusion: connective tissue diseases, vasculitides, sarcoidosis, nonmalignant eosinophilia-related disorders, adult-onset Still’s disease and other organ-specific autoimmune diseases with more than one organ involved.

### ECMO implantation

The detailed surgical procedure for femoral–femoral VA-ECMO or femoral–jugular VV-ECMO placement was described previously [7–9]. Briefly, trained cardiovascular surgeons performed all procedures in-ICU at bedside or in the cardiac angiography room because of patient’s hemodynamic instability. Femoral and/or jugular vessels were cannulated after limited cut-down using the Seldinger technique and, for VA-ECMO, an additional 7 French catheter was systematically inserted distally into the femoral artery to prevent severe leg ischemia. For highly unstable patients diagnosed with refractory cardiogenic shock or acute respiratory distress syndrome (ARDS) in other hospitals, our institution’s Mobile ECMO Retrieval Team traveled rapidly to primary-care hospitals with a portable ECMO system, installed the device before refractory multiorgan failure or ARDS occurred, and then transported the patient to our tertiary-care center [10].

### Study endpoints

The primary endpoint was in-hospital mortality, defined as death during the hospital stay consecutive to the first ICU admission and before the patient’s discharge to home. The secondary outcomes included ECMO weaning: bridge-to-recovery, bridge-to-transplantation (lung or cardiac) and bridge-to-long term ventricle assist device.

### Data collection

The following information was collected on standardized forms: epidemiological parameters; SRD clinical, biological and therapeutic history; clinical manifestations; laboratory findings; ECMO type, indication and complication(s); Survival after Veno-Arterial ECMO (SAVE) [11] and Respiratory Extracorporeal Membrane Oxygenation Survival Prediction (RESP) [12] scores, that are survival predictors in VA-ECMO and VV-ECMO patients, respectively; in-ICU treatments; organ-support treatments; SRD-specific treatments introduced in the ICU; ECMO-weaning status; bridge-to-transplantation or left ventricular assist device (LVAD); ICU complications; vital status, transplantation status at ICU and hospital discharges and at last follow-up.

### Statistical analyses

Results for categorical variables, expressed as number (%), were compared with *χ*^2^ tests; those for continuous variables, expressed as mean ± standard deviation or median [25–75th percentile interquartile range (IQR)], were compared using Student’s *t*-test or Wilcoxon’s rank test. Normality of continuous variable distribution was assessed with the Shapiro–Wilk test; when not normal, Wilcoxon’s rank test was used for comparisons.

First, patients’ characteristics (laboratory findings, in-ICU organ-failure treatment(s), SRD-specific manifestations and treatment(s), complications and outcomes) were subjected to descriptive analysis. Next, the mean/median values and frequencies of patients’ characteristics were compared according to the primary endpoint for the entire population and in the following subgroups: flare-related admission and VA/VV-ECMO. Then, for each subgroup, a Cox proportional hazards model, including the variables associated with the primary endpoint (entry threshold: *P* < 0.05), was run using backward-stepwise variable elimination (exit threshold: *P* > 0.10). Continuous variable were dichotomized to using the cut-offs with the best association with the primary endpoint in univariable Cox proportional hazards model. All potential explanatory variables included in the multivariable analyses were subjected to colinearity analysis with a correlation matrix. When colinearity was found (variance inflation factor > 5), only one of the two variables could be included the model. Statistical significance was defined as *P* < 0.05. Analyses were computed with IBM SPSS Statistics v22.0 software (IBM Corp, Armonk, NY).

### Ethical considerations

The database is registered with the “*Commission Nationale de l’Informatique et des Libertés*” (2217847v0). In accordance with the ethical standards of our hospital’s institutional review board, the Committee for the Protection of Human Subjects, and French law, written informed consent was not needed for demographic, physiological and hospital-outcome data analyses, because this observational study did not modify existing diagnostic or therapeutic strategies; however, patients were informed of their inclusion in the study.

## Results

### General characteristics

**Fig. 1:**
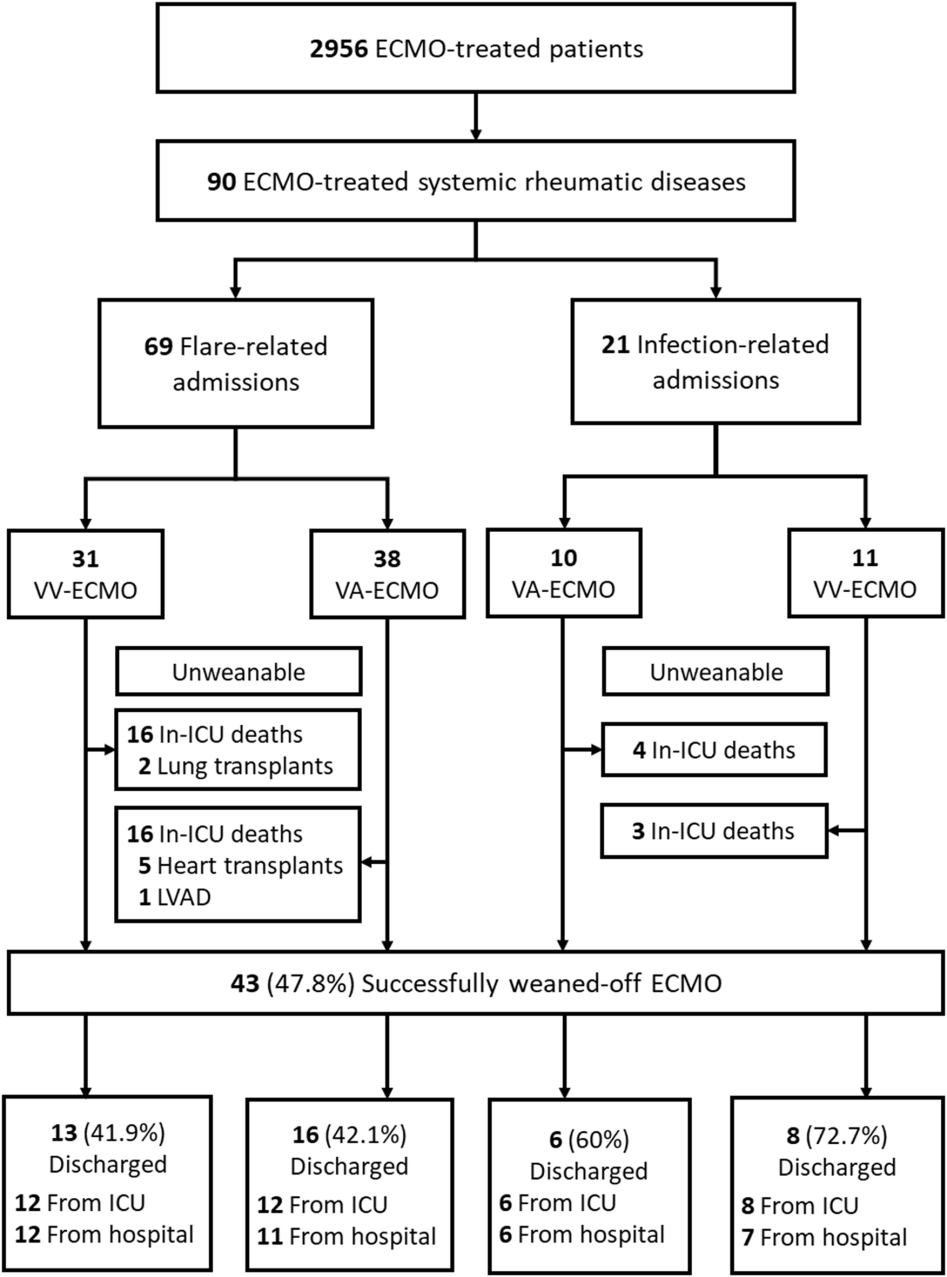
Flow chart of the 90 patients with systemic rheumatic disease requiring extracorporeal membrane oxygenation. *ICU* intensive care unit, *LVAD* left ventricular assist device, *VA/VV-ECMO* venoarterial/venovenous-extracorporeal membrane oxygenation

During the study period, 90 SRD patients requiring ICU admission (male/female ratio: 0.5; mean age at ICU admission: 41.6 ± 15.2 years) received VV-ECMO (*n* = 42, 46.7%) or VA-ECMO (*n* = 48, 53.3%). Their demographics and the SRD characteristics are detailed in Table 1 and Additional file 1: Table S1. SRD was diagnosed in-ICU for 34.4% patients. The main diagnoses were: connective tissue disease (57.8%), vasculitis (11.1%), rheumatic disorders (11.1%) and sarcoidosis (5.6%). The organs most frequently affected pre-admission were: lung (47.8%), joints (38.9%), skin, heart and kidney. Before ICU admission, 47.8% of the patients took corticosteroids regularly and 36.7% immunosuppressants. Three-quarters were admitted for an SRD flare and about one-quarter for an infection. The flow chart reports patients’ outcomes according to the reason for admission and ECMO hook-up (Fig. 1). In-ICU mortality, in-hospital mortality and in-hospital mortality/LVAD/ transplantation rates were: 48.9, 51.1 and 60.0%.

**Table 1:** Characteristics of 90 SRD patients given ECMO support

Variables	Value
Women	60 (66.7)
Body mass index, kg/m^2^	26.3 ± 6.8
Age at ICU admission, years	41.6 ± 15.2
Systemic rheumatic diseases	
Diagnosis in the ICU	31 (34.4)
Diagnosis-to-ICU interval*, months	93 (25–132)
Connective tissue diseases	52 (57.8)
Systemic lupus erythematosus	22 (24.4)
Idiopathic inflammatory myositis	12 (13.3)
Antiphospholipid syndrome	12 (13.3)
Systemic sclerosis	5 (5.6)
Mixed connective tissue disease	5 (5.6)
Sjögren’s syndrome	3 (3.3)
Rheumatic disorders	10 (11.1)
Vasculitides	10 (11.1)
Goodpasture’s syndrome	3 (3.3)
ANCA-associated	5 (5.6)
Small-vessel	1 (1.1)
IgA-associated	1 (1.1)
Sarcoidosis	5 (5.6)
Nonmalignant eosinophilia-related diseases	4 (4.4)
Adult-onset Still’s disease	4 (4.4)
Others^†^	5 (5.6)
Pre-ICU Specific treatment(s)	
Corticosteroids	43 (47.8)
Immunosuppressant(s)^§^	33 (36.7)
Flare-related admission	69 (76.7)
Infection-related admission	21 (23.3)

Continuous variables are expressed as mean ± standard deviation or median [interquartile range]; categorical variables are expressed as *n* (%)

*ICU* intensive care unit, *ANCA* antineutrophil cytoplasm antibodies, *ECMO* extracorporeal membrane oxygenation, *SRD* systemic rheumatic disease

^*^Three missing data

^†^One each: myasthenia gravis, neuromyelitis optica, multicentric Castleman’s disease, autoimmune thrombocytopenic purpura or inflammatory bowel disease

^§^Methotrexate *n* = 15, azathioprine *n* = 13, mycophenolate mofetil *n* = 9, cyclophosphamide *n* = 8, rituximab *n* = 6, tumor necrosis factor-inhibitor *n* = 6, calcineurin inhibitors *n* = 5, tocilizumab *n* = 2

The main ECMO complications were cannula-related infection, insertion-site hemorrhage and limb ischemia. In-ICU–acquired infections occurred in 65.6% of patients; their sites and pathogens are reported in Additional file 1: Table S2.

### Uni- and multivariable analyses of in-hospital mortality-associated factors

Nonsurvivors, compared to survivors, were less quickly admitted to the ICU after symptom onset and hospital admission, had more frequent SRD heart involvement before admission, higher day-0 SAPS II and SOFA scores, lower RESP and SAVE scores, and more frequently received vasopressors and renal replacement therapy in ICU (Table 2). Nonsurvivors also had more frequent in-ICU–acquired infections, especially fungal, and ECMO insertion-site hemorrhages. The frequencies of flare-related admissions, in-ICU SRD diagnoses and VA/VV-ECMO percentages were not different for the two groups.

**Table 2:** In-ICU characteristics and outcomes of the 90 ECMO-treated SRD patients: hospital survivors vs. nonsurvivors

Characteristic	*n*/n*	Survivors *n* = 44	Nonsurvivors *n* = 46	*P* value
Women		26 (59.1)	34 (73.9)	0.1
Age at admission, years		38.9 ± 15.6	44.2 ± 14.5	0.1
Symptom-onset-to-ICU interval, days		8 [3–21.5]	16 [6–41]	0.03
Hospital-to-ICU interval, days		3.5 [1–8]	8 [3–19]	0.007
Days in ICU		19 [9–59]	17 [5–37]	0.1
Pre-ICU–admission SRD				
Connective tissue diseases		23 (52.3)	29 (63.0)	0.3
Vasculitides		7 (15.9)	3 (6.5)	0.2
Corticosteroids		17 (38.6)	26 (56.5)	0.1
Immunosuppressant(s)		14 (31.8)	19 (41.3)	0.4
Heart involvement		7 (15.9)	16 (34.8)	0.04
In-ICU SRD				
Flare-related admission		31 (70.5)	38 (82.6)	0.2
In-ICU diagnosis		14 (31.8)	17 (37)	0.6
Corticosteroids		28 (63.6)	30 (65.2)	0.9
Immunosuppressant(s)		13 (29.5)	18 (39.1)	0.3
Organ failure at ICU admission				
Day-0 SAPS II		57 [45–66]	62 [48–75]	0.07
Day-0 SOFA score		12 [9–15]	15 [12–18]	0.01
ECMO				
VV-ECMO		21 (47.7)	21 (45.7)	0.8
RESP score		2 [− 0.5 to 3]	− 2 [− 4 to 0]	0.001
VA-ECMO		23 (52.3)	25 (54.3)	0.8
SAVE score		− 0.5 [− 6 to 1.7]	− 11 [− 15 to − 5]	0.001
Days on ECMO		8 [5–22.2]	8 [1–20.2]	0.2
ECMO complication				
Limb ischemia		6 (13.6)	5 (10.9)	0.7
Insertion-site hemorrhage		6 (13.6)	14 (30.4)	0.06
Cannula-related infection		10 (22.7)	10 (21.7)	0.9
In-ICU organ support				
Dobutamine		23 (52.3)	20 (43.5)	0.4
Vasopressors		38 (86.4)	45 (97.8)	0.04
Mechanical ventilation		42 (95.5)	45 (97.8)	0.5
Renal replacement therapy		18 (40.9)	31 (67.4)	0.01
Highest in-ICU value				
Arterial lactate, mmol/L	43/46	6.4 [3.2–10]	13 [5.9–18]	0.001
Troponin, ULN	39/45	13.2 [4.4–41.7]	10.7 [1.8–53.2]	0.5
Serum creatinine, µmol/L	42/41	143 [89–342]	147 [93–218]	0.8
Lowest in-ICU value				
Platelet count, G/L	43/46	56 [30–103]	23 [7–40]	< 0.0001
Prothrombin time, %	43/46	47 [37–60]	34 [16–55]	0.005
Outcome				
In-ICU–acquired infection		26 (59.1)	33 (71.7)	0.2
Fungal infection		3 (6.8)	11 (23.9)	0.02
Transplantation		8 (18.2)	1 (2.2)	0.01
Heart		3 (6.8)	1 (2.2)	n/a
Heart–kidney combined		1 (2.3)	0 (0)	n/a
Lung		4 (9.1)	0 (0)	n/a
Left ventricular assist device		1 (2.3)	1 (2.2)	0.9
Weaning		36 (81.8)	7 (15.2)	< 0.0001
In-ICU mortality		0 (0)	44 (95.7)	< 0.0001

Continuous variables are expressed as mean ± standard deviation or median [interquartile range] and compared with Student’s *t*-test or Wilcoxon’s rank test; categorical variables are expressed as *n* (%) and compared with *χ*^2^ tests

*ICU* intensive care unit, *LVEF* left ventricle ejection fraction, *RESP* Respiratory Extracorporeal Membrane Oxygenation Survival Prediction, *SAPS *Simplified Acute Physiology Score, *SAVE *Survival after Veno-Arterial ECMO, *SOFA* Sequential Organ-Failure Assessment, *SRD* systemic rheumatic disease, *VA-/VV-ECMO* venoarterial/venovenous-extracorporeal membrane oxygenation, *ULN* upper limit of normal value, *VTI* velocity–time integral

^*^Numbers of survivor/nonsurvivor data available

The Cox proportional hazards model univariable and multivariable analyses for the 90 SRD patients (Table 3) retained: pre-admission SRD heart involvement; day-0 SAPS II score ≥ 70; arterial lactate ≥ 7.5 mmol/L and bilirubin ≥ 125 µmol/L, as independently associated with in-hospital mortality.

**Table 3:** Univariable and multivariable analyses of factors associated with in-hospital mortality for the 90 ECMO-treated SRD patients

Factor	Univariable analysis			multivariable analysis		
	HR	95% CI	*P* value	HR	95% CI	*P* value
Age ≥ 40 years	1.4	0.8–2.5	0.3			
Women	1.5	0.8–2.9	0.2			
Pre-admission SRD lung involvement	0.5	0.3–0.9	0.04	0.8	0.4–1.6	0.6
Pre-admission SRD heart involvement	1.7	0.9–3.2	0.08	**2.9**	**1.5–5.8**	**0.001**
Corticosteroids before admission	1.7	0.9–3.1	0.07	1.8	0.9–3.3	0.052
Immunosuppressants before admission	1.3	0.7–2.3	0.4			
In-ICU SRD diagnosis	1.0	0.6–1.9	0.9			
Day-0 SAPS II ≥ 70	2.7	1.5–4.9	0.001	**2.7**	**1.4–5.1**	**0.003**
Day-0 SOFA score ≥ 16	2.8	1.6–5.1	< 0.0001			
Flare-related admission	1.4	0.6–3.0	0.7			
VA-ECMO	1.3	0.7–2.4	0.3			
In-ICU corticosteroids	0.8	0.4–1.5	0.5			
In-ICU immunosuppressant(s)	1.0	0.5–1.8	0.9			
Vasopressors	5.3	0.7–38.6	0.1	2.7	0.3–21.0	0.3
Mechanical ventilation	1.8	0.2–13.2	0.5			
Renal replacement therapy	2.2	1.2–4.0	0.01	0.6	0.2–1.4	0.2
ICU-acquired infection	1.0	0.6–2.0	0.9			
Highest in-ICU value						
Arterial lactate ≥ 7.5 mmol/L	3.2	1.7–5.9	< 0.0001	**2.8**	**1.4–5.3**	**0.002**
Bilirubin ≥ 125 µmol/L	2.3	1.2–4.3	0.007	**2.0**	**1.0–3.9**	**0.04**
Lowest in-ICU value						
Platelet count < 50 G/L	2.9	1.4–6.0	0.004	1.8	0.7–4.5	0.2

Bold values indicates statistically significant in multivariable analysis

The multiple Cox proportional hazards model used backward-stepwise variable elimination (with variable exit threshold set at *P* > 0.10). All potential explanatory variables included in the multivariable analyses were subjected to colinearity analysis with a correlation matrix. Variables associated with one another were not included in the model**.** Statistical significance was defined as *P* < *0*.05

*ICU* intensive care unit, *SAPS* Simplified Acute Physiology Score, *SOFA* sequential organ failure assessment, *SRD* systemic rheumatic diseases, *VA-ECMO* venoarterial-extracorporeal membrane oxygenation

### Uni- and multivariable analyses of in-hospital mortality-associated factors: flare-related admissions

Among the 69 flare-related admissions: 44.9% patients received VV-ECMO and 55.1% VA-ECMO, 21 could be weaned-off ECMO and 10 were bridged-to-transplant (*n* = 8) or -LVAD (*n* = 2). Nonsurvivors, compared to survivors, had more frequent SRD heart involvement before admission, higher day-SOFA scores, lower RESP and SAVE scores, and more frequently received vasopressors and renal replacement therapy in ICU (Table 4).

**Table 4:** In-ICU characteristics and outcomes of the 69 ECMO-treated SRD flare patients: hospital survivors vs. nonsurvivors

Characteristic	*n*/n*	Survivors *n* = 31	Nonsurvivors *n* = 38	***P value***
Women		16 (51.6)	27 (71.1)	0.1
Age at admission, years		37.6 ± 16.2	44.5 ± 14.6	0.07
Symptom-onset-to-ICU interval, days		8 [3–23]	18 [7–42]	0.02
Hospital-to-ICU interval, days		3 [1–8]	9 [4–22.2]	0.002
Days in ICU		19 [9–77]	17 [5–36]	0.1
Pre-admission SRD				
Connective tissue diseases		15 (48.4)	34 (89.5)	0.2
Vasculitides		6 (19.4)	3 (7.9)	0.2
Corticosteroids		10 (32.3)	19 (50)	0.1
Immunosuppressant(s)		7 (22.6)	13 (34.2)	0.3
Heart involvement		6 (19.4)	16 (42.1)	0.04
In-ICU SRD				
In-ICU diagnosis		12 (38.7)	17 (44.7)	0.6
Corticosteroids		24 (77.4)	29 (76.3)	0.9
Immunosuppressant(s)		12 (38.7)	18 (47.4)	0.5
Organ failures at ICU admission				
Day-0 SAPS II		55 [30–66]	62 [48–73]	0.06
Day-0 SOFA score		12 [8–14]	15 [12–18]	0.006
ECMO				
VV-ECMO		14 (45.2)	17 (44.7)	0.9
RESP score		1.5 [− 0.2 to 3]	− 2 [− 4 to 0]	0.007
VA-ECMO		17 (54.8)	21 (55.3)	0.9
SAVE score		0 [− 6 to 1]	− 8.5 [− 13.7 to − 3.5]	0.001
Days on ECMO		48 [7–73]	11 [4–29]	0.2
ECMO complications				
Limb ischemia		3 (9.7)	3 (7.9)	0.8
Insertion-site hemorrhage		5 (16.1)	10 (26.3)	0.1
Cannula-related infection		8 (25.8)	7 (18.4)	0.5
In-ICU organ support				
Dobutamine		18 (58.1)	16 (42.1)	0.2
Vasopressors		25 (80.6)	37 (97.4)	0.02
Mechanical ventilation		29 (93.5)	37 (97.4)	0.4
Renal replacement therapy		11 (35.5)	24 (63.2)	0.02
Highest in-ICU value				
Arterial lactate, mmol/L		5.5 [3.1–8.8]	13.1 [5.4–18.2]	0.004
Troponin, ULN	28/37	15.6 [5.3–84.4]	10.3 [0.7–37.9]	0.1
Serum creatinine, µmol/L	30/34	118 [89–342]	149 [93–217]	0.7
Lowest in-ICU value				
Platelet count, G/L		56 [30–103]	25 [9–38]	< 0.0001
Prothrombin time, %		43 [35–60]	34 [16–55]	0.03
Outcome				
In-ICU–acquired infection		19 (61.3)	28 (73.7)	0.2
Fungal infection		3 (9.7)	9 (23.7)	0.1
Transplantation		7 (22.6)	1 (2.6)	0.01
Heart		2 (6.5)	1 (2.6)	n/a
Heart–kidney combined		1 (3.2)	0 (0)	n/a
Lung		4 (12.9)	0 (0)	n/a
Left ventricular assist device		1 (3.2)	1 (2.6)	0.9
Weaning		23 (74.2)	6 (15.8)	< 0.0001
In-ICU mortality		0 (0)	37 (97.4)	< 0.0001

Continuous variables are expressed as mean ± standard deviation or median [interquartile range] and were compared with Student’s *t*-test or Wilcoxon’s rank test; categorical variables are expressed as *n* (%) and were compared with *χ*^2^ tests

*ICU* intensive care unit, *LVEF* left ventricle ejection fraction, *RESP* Respiratory Extracorporeal Membrane Oxygenation Survival Prediction, *SAPS *Simplified Acute Physiology Score, *SAVE *Survival after Veno-Arterial ECMO, *SOFA* Sequential Organ-Failure Assessment, *SRD* systemic rheumatic disease, *ULN* upper limit of normal value, *VA-/VV-ECMO* venoarterial/venovenous extracorporeal membrane oxygenation, *VTI *velocity–time integral

^*^Numbers of survivor/nonsurvivor data available

Similarly to the whole cohort, the Cox proportional hazards model univariable and multivariable analyses (Table 5) retained: pre-admission SRD heart involvement, day-0 SAPS II score ≥ 70, arterial lactate ≥ 7.5 mmol/L and bilirubin ≥ 125 µmol/L, as independently associated with in-hospital mortality.

**Table 5:** Univariable and multivariable analyses of factors associated with in-hospital mortality of the 69 ECMO-treated SRD flare patients

Factor	Univariable analysis			Multivariable analysis		
	HR	95% CI	*P* value	HR	95% CI	*P* value
Age ≥ 40 years	1.5	0.7–2.8	0.2			
Women	1.7	0.8–3.4	0.1			
Pre-admission SRD lung involvement	0.6	0.3–1.1	0.08	0.7	0.4–1.5	0.4
Pre-admission SRD heart involvement	1.8	0.9–3.5	0.07	**2.9**	**1.4–6.0**	**0.003**
In-ICU SRD diagnosis	1.1	0.6–2.0	0.8			
Pre-admission corticosteroids	1.7	0.9–3.1	0.1			
Pre-admission immunosuppressant(s)	1.5	0.8–2.9	0.2			
Day-0 SAPS II ≥ 70	2.4	1.2–4.7	0.01	**3.1**	**1.5–6.5**	**0.002**
Day-0 SOFA ≥ 16	2.9	1.5–5.6	0.001			
Symptom-onset-to-ICU interval ≥ 10 days	1.6	0.8–3.1	0.2			
VA-ECMO	1.2	0.6–2.2	0.6			
In-ICU corticosteroids	0.7	0.4–1.6	0.5			
In-ICU immunosuppressant(s)	0.9	0.5–1.7	0.8			
Vasopressors	5.9	0.8–43.0	0.08	2.8	0.3–21.7	0.3
Mechanical ventilation	1.9	0.3–14.2	0.5			
Renal replacement therapy	2.1	1.1–4.0	0.03	0.5	0.2–1.4	0.2
ICU-acquired infection	1.0	0.5–2.2	0.9			
Highest In-ICU value						
Arterial lactate ≥ 7.5 mmol/L	2.7	1.4–5.2	0.004	**2.7**	**1.3–5.3**	**0.006**
Bilirubin ≥ 125 µmol/L	2.2	1.1–4.5	0.02	**2.4**	**1.1–4.9**	**0.02**
Lowest in-ICU value						
Platelet count < 50 G/L	3.2	1.3–7.7	0.009	1.5	0.6–4.6	0.4

Bold values indicates statistically significant in multivariable analysis

The multiple Cox proportional hazards model used backward-stepwise variable elimination (with the variable exit threshold set at *P* > 0.10). All potential explanatory variables included in the multivariable analyses were subjected to colinearity analysis with a correlation matrix. Variables associated with one another were not included in the model. Statistical significance was defined as *P* < *0*.05

*ICU* intensive care unit, *SAPS* Simplified Acute Physiology Score, *SOFA* Sequential Organ Failure Assessment, *SRD* systemic rheumatic diseases, *VA-ECMO* venoarterial-extracorporeal membrane oxygenation

### Uni- and multivariable analyses of VA-ECMO–associated in-hospital mortality factors

Among the 48 VA-ECMO patients, 23 (47.9%) survived to hospital discharge. Nonsurvivors, compared to survivors, more frequently had SRD heart involvement before admission, higher day-0 SAPS II and SOFA scores, lower left ventricular ejection fraction before cannulation and SAVE scores, more frequently received in-ICU vasopressors and renal replacement therapy, and more frequently experienced in-ICU cardiac arrest (Additional file 1: Table S3).

The Cox proportional hazards model univariable and multivariable analyses for the 48 patients given VA-ECMO support (Additional file 1: Table S4) retained: in-ICU SRD diagnosis, day-0 SAPS II score ≥ 70 and arterial lactate ≥ 7.5 mmol/L as independently associated with in-hospital mortality.

### Uni- and multivariable analyses of VV-ECMO–associated in-hospital mortality-associated factors

Among the 42 patients receiving VV-ECMO, 21 (50.0%) survived to hospital discharge. Nonsurvivors, compared to survivors, were less quickly admitted to the ICU after symptom onset and hospital admission; had vasculitis less frequently, lower RESP scores and more in-ICU–acquired infections, especially ventilator-associated pneumonia and invasive fungal infection (Additional file 1: Table S5).

The Cox proportional hazards model univariable and multivariable analyses for these 42 patients (Additional file 1: Table S6) retained: vasculitis, day-0 SAPS II score ≥ 70, ICU-acquired ventilator-associated pneumonia and arterial lactate ≥ 7.5 mmol/L, as independently associated with in-hospital mortality.

## Discussion

SRDs are heterogeneous diseases, whose severe organ involvement may lead to end-organ failure requiring ICU admission. Their rarity makes diagnoses sometimes difficult and management of critically ill patients a delicate undertaking. When end-organ lung or heart failure occurs, the capacity to recover is uncertain, especially for chronic SRD involvement, even with the latest therapeutic innovations. ECMO is an emerging rescue therapy, whose indications for VV [13] or VA hook-up [14] have not yet been clearly delineated. Data are urgently awaited to support or refute the indication of ECMO for SRD patients.

Herein, we report the largest series of ECMO-treated, severely ill SRD patients. Available literature is scarce, other than multiple case reports, and ECMO use was anecdotical in previous populations: 6 (1.6%) patients in the study by Dumas and colleagues [2], 6 (7.3%) and 3 (3.1%) in the largest ICU studies on antineutrophil cytoplasm antibody-associated vasculitides [15, 16]. A significant number of the 62 (11.8%) ECMO-treated patients in Larcher and colleagues’ recent paper [3] were managed in our center and are also included herein, however this study did not specifically addressed the characteristics, management and outcomes of ECMO-treated patients and, therefore, does not duplicate the results of the present study.

Our analyses identified several new findings. Unlike previous studies, most of our patients were admitted for an SRD flare and only a quarter for an infection. This inverse proportion reflects bias related to the population for which ECMO is indicated: a small percentage of bacterial/viral pneumonias require VV-ECMO implantation and few infections (mainly severe septic shock) will need VA-ECMO cannulation. At the same time, the number of patients admitted for their first SRD manifestation was particularly high: one-third of our patients vs. one-tenth in previous reports [2, 3]. While those admissions for infection had usually been associated with worse outcomes, the in-hospital survival rates of our flare and infection patients were similar. Some classical, ICU-prognostic factors were not associated with in-hospital mortality, particularly: age, mechanical ventilation, vasopressor use and renal replacement therapy. That finding probably reflects the stringent selection of our patients and the very high level of in-ICU organ support that most of them received.

Our in-hospital mortality was significantly higher than previously reported. Indeed, the main series of critically ill SRD patients reported 16–21% in-ICU [2, 4] and 20–43% in-hospital–mortality rates [1, 3, 17–21]. However, our patients were obviously more severely ill, as shown by their higher median day-0 SOFA scores and day-0 SAPS II, respectively: 13.5 vs. 5–7.2 [2, 3, 19] and 59 vs. 29–45 [3, 4, 21]. Moreover, our in-hospital–mortality rate was similar to those of ESLO patients: ~ 43% VV-ECMO–treated [12] and ~ 58% VA-ECMO–treated [11].

Nine (10%) patients with refractory heart (*n* = 5) or lung (*n* = 4) failure could be successfully bridged to emergency transplantation. While urgent cardiac transplantation in patients under VA-ECMO is frequent, lung transplantation of unlisted patients on VV-ECMO is unusual. We advocate that SRD patients, especially young patients, who could not be weaned-off ECMO, should be considered for heart or lung transplantation.

SRD diagnosis, corticosteroid and immunosuppressant use before ICU admission or thereafter were not associated with in-hospital mortality for the entire population. However, specific SRD heart involvement known before ICU admission was associated with poorer outcomes, independently of VA-ECMO cannulation. That finding underlines the impact of heart sequalae from previous SRD flares on these patients’ prognoses. Conversely, patients receiving VA-ECMO support for the first SRD manifestation had poorer outcomes, underscoring the severity of SRD myocardial involvement.

Importantly, for the VV-ECMO–treated subgroup, a vasculitis diagnosis was strongly and independently associated with favorable outcomes. Their intra-alveolar hemorrhages were usually quickly reversible under specific regimens combining corticosteroids, rituximab/cyclophosphamide and plasma exchanges. Our results strongly support the use of VV-ECMO for these patients.

Corticosteroid and immunosuppressant administration can be associated with in-ICU–acquired infection, especially for patients on ECMO. Indeed, our series’ infection frequency was high, but rates were similar for infection vs. flare admissions, despite the latter having more frequently received corticosteroids and immunosuppressant(s). The rates of ventilator-associated pneumonia and bloodstream infections were in accordance with those in the ESLO database for VV-ECMO–treated patients [22]. Invasive fungal infections were particularly high (15%) and ventilator-associated pneumonia was independently associated with in-hospital mortality of VV-ECMO–treated patients, suggesting that careful attention should be paid to infectious complications in these profoundly immunosuppressed patients.

Our study has limitations and strengths. First, despite its retrospective, observational design, many patients had rare diseases requiring a still evolving and relatively rarely used rescue technique. Second, patient inclusion lasted > 14 years, meaning inevitable heterogeneity of diagnoses and management, but most patients were included during the last decade. Third, it is likely that ECMO support was declined for some SRD patients that were considered unfit to endure such an aggressive technique. The mortality rates herein reported should, therefore, be extrapolated with caution as they refer to an highly selected population of patients. Lastly, the main analysis considered VA- and VV-ECMO patients jointly. The reasons for ICU admission and ECMO cannulation, and the characteristics, management and outcomes of these patients obviously differ. We acknowledge that such an analysis might confound the results and their interpretation. However, the analysis aimed to present a comprehensive, real-life picture of ECMO treatment of SRD patients, with separate analyses of VA- and VV-ECMO subgroups thereafter.

## Conclusion

ECMO is a relevant rescue technique for critically ill SRD patients, with 49% survival to hospital discharge. Vasculitis was independently associated with a favorable outcome of VV-ECMO–cannulated patients. Further studies are needed to specify the role of ECMO for SRD patients.

## Supplementary Information

**Additional file1**: **Table S1**. Supplementary characteristics of 90 SRD Patients Given ECMO Support. **Table S2**. Microbiological Findings for In-ICU–Acquired Infections of the 90 ECMO-Treated SRD Patients. **Table S3**. In-ICU Characteristics and Outcomes of the 48 VA-ECMO–Treated SRD Patients: Hospital Survivors vs. Nonsurvivors. **Table S4**. Univariable and Multivariable Analyses of Factors Associated with In-Hospital Mortality for the 48 VA-ECMO–Treated SRD Patients. **Table S5**. In-ICU Characteristics and Outcomes of the 42 VV-ECMO–Treated SRD Patients: Hospital Survivors vs. Nonsurvivors. **Table S6**. Univariable and Multivariable Analyses of Factors Associated with In-Hospital Mortality for the 42 VV-ECMO–Treated SRD Patients (DOCX 58 KB)

## Data Availability

All data generated or analyzed during the study are included in this published article and the its supplementary information files.

## Abbreviations

ARDS: Acute respiratory distress syndrome
CI: Confidence interval
HR: Hazard ratio
ICU: Intensive care unit
IQR: 25–75Th percentile interquartile range
LVAD: Left ventricle assist device
RESP: Respiratory Extracorporeal Membrane Oxygenation Survival Prediction
SAPS: Simplified Acute Physiology Score
SAVE: Survival after Veno-Arterial ECMO
SOFA: Sequential Organ-Failure Assessment
SRD: Systemic Rheumatic Diseases
VA-ECMO: Venoarterial-extracorporeal membrane oxygenation
VV-ECMO: Venovenous extracorporeal membrane oxygenation
