# Supplementary material for: Outcomes of severe systemic rheumatic disease patients requiring extracorporeal membrane oxygenation
Source: Ann Intensive Care. 2021 Feb 9;11:29. doi: 10.1186/s13613-021-00819-3 (PMC7871308; doi:10.1186/s13613-021-00819-3)
Supplement: Supplementary file 1 — Additional file1: Table S1. Supplementary characteristics of 90 SRD Patients Given ECMO Support. Table S2. Microbiological Findings for In-ICU–Acquired Infections of the 90 ECMO-Treated SRD Patients. Table S3. In-ICU Characteristics and Outcomes of the 48 VA-ECMO–Treated SRD Patients: Hospital Survivors vs. Nonsurvivors. Table S4. Univariable and Multivariable Analyses of Factors Associated with In-Hospital Mortality for the 48 VA-ECMO–Treated SRD Patients. Table S5. In-ICU Characteristics and Outcomes of the 42 VV-ECMO–Treated SRD Patients: Hospital Survivors vs. Nonsurvivors. Table S6. Univariable and Multivariable Analyses of Factors Associated with In-Hospital Mortality for the 42 VV-ECMO–Treated SRD Patients (DOCX 58 KB) [file 13613_2021_819_MOESM1_ESM.docx]

| **e-Table E1.** Supplementary characteristics of 90 SRD Patients Given ECMO Support | |
| --- | --- |
| **Variables** | **Value** |
| Pre-ICU organ involvement |  |
| Lungs | 43 (47.8) |
| Joints | 35 (38.9) |
| Skin | 27 (30) |
| Heart | 23 (25.6) |
| Kidneys | 18 (20) |
| Central nervous system | 13 (14.4) |
| Muscles | 12 (13.3) |
| Others^‡^ | 33 (36.7) |
| Pre-ICU Specific treatment(s) |  |
| Corticosteroids | 43 (47.8) |
| Immunosuppressant(s)^§^ | 33 (36.7) |
| Hydroxychloroquine | 13 (14.4) |
| Anticoagulant | 7 (7.8) |
| Intravenous immunoglobulins | 5 (5.6) |
| Plasma exchange | 3 (3.3) |
| Others^¶^ | 4 (4.4) |
| Flare-related admission | 69 (76.7) |
| Flare-related organ involvement |  |
| Heart | 33/69 (47.8) |
| Lungs | 40/69 (58) |
| Macrophage-activation syndrome | 10/69 (14.5) |
| Kidneys | 9/69 (13.0) |
| Central nervous system | 4/69 (5.8) |
| Hematological | 2/69 (2.9) |
| Liver | 2/69 (2.9) |
| Gastrointestinal tract | 1/69 (1.4) |
| Infection-related admission | 21 (23.3) |
| Community-acquired pneumonia | 9 (42.9) |
| Virus-related pneumonia | 6 (28.6) |
| *Pneumocystis* pneumonia | 2 (9.5) |
| Abdominal | 1 (4.8) |
| Others^║^ | 5 (23.8) |
| *Definition of abbreviations:* ICU = intensive care unit.  Continuous variables are expressed as mean±standard deviation or median [interquartile range]; categorical variables are expressed as *n* (%).  ^‡^Hematological *n*=9, ear-nose-throat and eye *n*=9, liver *n*=4, thrombosis *n*=3, obstetrical *n*=3, gastrointestinal tract *n*=2.  ^§^Methotrexate *n*=15, azathioprine *n*=13, mycophenolate mofetil *n*=9, cyclophosphamide *n*=8, rituximab *n*=6, tumor necrosis factor-inhibitor *n*=6, calcineurin inhibitors *n*=5, tocilizumab *n*=2.  ^¶^Leflunomide *n*=2, eculizumab *n*=1, chemotherapy (R-CHOP) *n*=1.  Viral myocarditis *n*=2, endocarditis *n*=1, catheter-related infection *n*=1, streptococcal toxic-shock syndrome *n*=1. | |

| **e-Table E2.** Microbiological Findings for In-ICU–Acquired Infections of the 90 ECMO-Treated SRD Patients | | | | | | | | |
| --- | --- | --- | --- | --- | --- | --- | --- | --- |
| **Ventilator-associated pneumonia, *n* (%)** | | **Bloodstream infection, *n* (%)** | | **Cannula/catheter-related infection, *n* (%)** | | **Others*****, *n* (%)** | |  |
| Patients | 45 (50%) | Patients | 16 (17.8%) | Patients | 23 (25.6%) | Patients | 12 (13.3%) |  |
| Episodes, *n* | 84 | Episodes, *n* | 19 | Episodes, *n* | 23 | Episodes, *n* | 15 |  |
| Organism, *n* | 97 | Organism, *n* | 21 | Organism, *n* | 33 | Organism, *n* | 18 |  |
| *Pseudomonas aeruginosa* | 39 (40.2%) | *Candida* spp. | 8 (38.1%) | *Enterococcus* spp. | 6 (18.2%) | *Cytomegalovirus* | 5 (27.8%) |  |
| *Klebsiella spp.* | 9 (9.3%) | *Enterococcus* spp. | 4 (19.0%) | *Pseudomonas aeruginosa* | 5 (15.2%) | *Candida* spp. | 2 (11.1%) |  |
| Herpes Simplex Virus | 8 (8.2%) | *Pseudomonas aeruginosa* | 3 (14.3%) | *Escherichia coli* | 5 (15.2%) | *Enterococcus* spp. | 2 (11.1%) |  |
| *Escherichia coli* | 5 (5.2%) | *Escherichia coli* | 1 (4.8%) | *Enterobacter* spp. | 3 (9.1%) | *Enterobacter* spp*.* | 2 (11.1%) |  |
| *Staphylococcus aureus* | 4 (4.1%) | *Enterobacter* spp. | 1 (4.8%) | *Klebsiella* spp. | 3 (9.1%) | *Pseudomonas aeruginosa* | 1 (5.6%) |  |
| *Enterococcus* spp. | 4 (4.1%) | *Streptococcus* spp. | 1 (4.8%) | *Proteus* spp. | 3 (9.1%) | *Escherichia coli* | 1 (5.6%) |  |
| *Enterobacter* spp. | 4 (4.1%) | *Proteus* spp. | 1 (4.8%) | *Candida* spp. | 2 (6.1%) | Herpes Simplex Virus | 1 (5.6%) |  |
| Stenotrophomonas *maltophilia* | 4 (4.1%) | CNS | 1 (4.8%) | *Staphylococcus aureus* | 2 (6.1%) | Varicella Zoster Virus | 1 (5.6%) |  |
| *Acinetobacter* spp. | 4 (4.1%) | *Geotrichum capitatum* | 1 (4.8%) | CNS | 1 (3.0%) | *Proteus* spp. | 1 (5.6%) |  |
| *Aspergillus* spp. | 4 (4.1%) |  |  | *Acinetobacter* spp. | 1 (3.0%) | CNS | 1 (5.6%) |  |
| *Streptococcus* spp. | 3 (3.1%) |  |  | Polymicrobial flora | 1 (3.0%) | *Acinetobacter* spp*.* | 1 (5.6%) |  |
| *Haemophilus influenzae* | 3 (3.1%) |  |  | *Streptococcus* spp. | 1 (3.0%) |  |  |  |
| *Proteus* spp. | 2 (2.1%) |  |  |  |  |  |  |  |
| Polymicrobial flora | 2 (2.1%) |  |  |  |  |  |  |  |
| CNS | 1 (1.0%) |  |  |  |  |  |  |  |
| *Candida* spp. | 1 (1.0%) |  |  |  |  |  |  |  |
| CNS = coagulase-negative staphylococci, ECMO = extracorporeal membrane oxygenation; ICU = intensive care unit; SRD = systemic rheumatic disease.  *One each: colitis, ilio-psoas hematoma, perineal cellulitis, left ventricular assist device drive or mediastinitis; two each: peritonitis or skin infection; six viral reactivations. | | | | | | | | |

| **e-Table E3.** In-ICU Characteristics and Outcomes of the 48 VA-ECMO–Treated SRD Patients: Hospital Survivors vs. Nonsurvivors | | | | |
| --- | --- | --- | --- | --- |
| **Characteristic** | ***n*/*n**** | **Survivors**  ***n*=23** | **Nonsurvivors**  ***n*=25** | ***P***  **Value** |
| Women |  | 14 (60.9) | 20 (80) | 0.1 |
| Age at admission, years |  | 39.1±14.6 | 41.2±13.6 | 0.6 |
| Symptom-onset-to-ICU interval, days |  | 7 [3–15] | 10 [3–25] | 0.4 |
| Hospital-to-ICU interval, days |  | 2 [1–6] | 5 [1–10] | 0.4 |
| Days in ICU |  | 10 [8–26] | 16 [1–37] | 0.5 |
| Pre-admission SRD |  |  |  |  |
| Connective tissue diseases |  | 14 (60.9) | 17 (68) | 0.6 |
| Vasculitides |  | 0 (0) | 1 (4) | 0.3 |
| Corticosteroids |  | 9 (39.1) | 13 (52) | 0.4 |
| Immunosuppressant(s) |  | 8 (34.8) | 8 (32) | 0.8 |
| Lung involvement |  | 8 (34.8) | 4 (16) | 0.1 |
| Heart involvement |  | 6 (26.1) | 14 (56) | 0.04 |
| In-ICU SRD |  |  |  |  |
| Flare-related admission |  | 17 (73.9) | 21 (84) | 0.4 |
| Diagnosis in the ICU |  | 3 (13.0) | 9 (36) | 0.07 |
| Corticosteroids |  | 12 (52.2) | 14 (56) | 0.8 |
| Immunosuppressant |  | 4 (17.4) | 9 (36) | 0.1 |
| Organ failures at ICU admission |  |  |  |  |
| Day-0 SAPS II |  | 55 [24–65] | 62 [44–77] | 0.04 |
| Day-0 SOFA score |  | 13 [9–16] | 18 [12–19] | 0.02 |
| VA-ECMO |  |  |  |  |
| LVEF before VA-ECMO, % | 23/20 | 15 [10–25] | 10 [5–20] | 0.08 |
| SAVE score | 19/23 | –0.5 [–6 to 1.7] | –11 [–15 to –5] | 0.001 |
| Days on ECMO |  | 7 [2–136] | 8 [1–20] | 0.6 |
| ECMO complications |  |  |  |  |
| Limb ischemia |  | 5 (21.7) | 4 (16) | 0.6 |
| Insertion-site hemorrhage |  | 4 (17.4) | 8 (32) | 0.2 |
| Cannula-related infection |  | 6 (26.1) | 5 (20) | 0.6 |
| In-ICU organ support |  |  |  |  |
| Dobutamine |  | 21 (91.3) | 18 (72) | 0.09 |
| Vasopressors |  | 20 (87) | 25 (100) | 0.06 |
| Mechanical ventilation |  | 21 (91.3) | 25 (100) | 0.1 |
| Renal replacement therapy |  | 8 (34.8) | 16 (64) | 0.04 |
| Highest in-ICU value |  |  |  |  |
| Arterial lactate, mmol/L | 22/25 | 7.1 [4.8–10.3] | 13.7 [6.9–19.5] | 0.008 |
| Troponin, ULN | 22/25 | 40 [11.9–93.3] | 25.0 [8.6–106.0] | 0.6 |
| Serum creatinine, µmol/L | 21/23 | 148 [106–309] | 159 [116–237] | 0.7 |
| Lowest in-ICU value |  |  |  |  |
| LVEF, % | 21/23 | 10 [10–10] | 10 [5–10] | 0.5 |
| Aortic VTI, cm | 19/21 | 6 [2–7] | 3 [0–6.5] | 0.08 |
| Platelet count, G/L | 22/25 | 38 [23–94] | 13 [6–34] | 0.002 |
| Prothrombin time, % | 22/25 | 43 [36–60] | 21 [14–52] | 0.03 |
| Outcome |  |  |  |  |
| In-ICU–acquired infection |  | 14 (60.9) | 15 (60) | 0.9 |
| Viral infection |  | 6 (26.1) | 1 (4) | 0.03 |
| Fungal infection |  | 1 (4.3) | 4 (16) | 0.2 |
| Transplantation |  | 6 (26.1) | 1 (4) | 0.03 |
| Heart |  | 3 (13.0) | 1 (4) | n/a |
| Heart–kidney combined |  | 1 (4.3) | 0 (0) | n/a |
| Lung |  | 2 (8.7) | 0 (0) | n/a |
| Left ventricular assist device |  | 1 (4.3) | 1 (4) | 0.9 |
| Weaning |  | 17 (73.9) | 5 (20) | <0.0001 |
| In-ICU mortality |  | 0 (0) | 24 (96) | <0.0001 |
| *Definition of abbreviations:* ICU = intensive care unit; LVEF = left ventricular ejection fraction; SAPS = Simplified Acute Physiology Score; SAVE = Survival after Veno-Arterial ECMO; SOFA = Sequential Organ Failure Assessment; SRD = systemic rheumatic disease; ULN = upper limit of normal value; VA-ECMO = venoarterial- extracorporeal membrane oxygenation; VTI = velocity-time integral.  Continuous variables are expressed as mean±standard deviation or median [interquartile range] and were compared with Student’s *t*-test or Wilcoxon’s rank test; categorical variables are expressed as *n* (%) and were compared with χ^2^ tests.  *Numbers of survivor/nonsurvivor data available. | | | | |

| **eTable E4.** Univariable and Multivariable Analyses of Factors Associated with In-Hospital Mortality for the 48 VA-ECMO–Treated SRD Patients | | | | | | | |
| --- | --- | --- | --- | --- | --- | --- | --- |
|  | **Univariable Analysis** | | |  | **Multivariable Analysis** | | |
| **Factor** | **HR** | **95% CI** | ***P* Value** |  | **HR** | **95% CI** | ***P* Value** |
| Age ≥40 yr | 1.4 | 0.6–3.1 | 0.4 |  |  |  |  |
| Women | 1.7 | 0.6–4.6 | 0.3 |  |  |  |  |
| Pre-ICU admission lung SRD involvement | 0.5 | 0.2–1.4 | 0.2 |  |  |  |  |
| Pre-admission SRD heart involvement | 1.6 | 0.7–3.6 | 0.2 |  |  |  |  |
| Pre-admission corticosteroids | 1.6 | 0.7–3.6 | 0.2 |  |  |  |  |
| Pre-admission Immunosuppressant(s) | 0.9 | 0.4–2.1 | 0.8 |  |  |  |  |
| In-ICU SRD diagnosis | 2.0 | 0.9–4.5 | 0.1 |  | **4.3** | **1.7–10.9** | **0.002** |
| Day-0 SAPS II ≥70 | 3.1 | 1.4–7.0 | 0.006 |  | **4.2** | **1.8–10.2** | **0.001** |
| Day-0 SOFA score ≥16 | 2.7 | 1.2–6.1 | 0.01 |  |  |  |  |
| Flare-related admission | 0.9 | 0.3–2.8 | 0.9 |  |  |  |  |
| In-ICU corticosteroids | 0.9 | 0.4–1.9 | 0.7 |  |  |  |  |
| In-ICU immunosuppressant(s) | 1.1 | 0.5–2.6 | 0.8 |  |  |  |  |
| Vasopressors | 23.9 | 0.1–>100 | 0.3 |  |  |  |  |
| Mechanical ventilation | 22.5 | 0.1->100 | 0.4 |  |  |  |  |
| Renal replacement therapy | 2.1 | 0.9–4.7 | 0.08 |  | 0.9 | 0.3–2.4 | 0.8 |
| ICU-acquired infection | 0.6 | 0.3–1.4 | 0.3 |  |  |  |  |
| Highest in-ICU value |  |  |  |  |  |  |  |
| Arterial lactate ≥7.5 mmol/L | 2.8 | 1.1–6.7 | 0.02 |  | **3.9** | **1.5–10.0** | **0.004** |
| Bilirubin ≥125 µmol/L | 1.6 | 0.6–3.7 | 0.3 |  |  |  |  |
| Lowest in-ICU value |  |  |  |  |  |  |  |
| Platelet count <50 G/L | 1.6 | 0.6–4.2 | 0.4 |  |  |  |  |

*Definition of abbreviations:* ICU = intensive care unit; SAPS = Simplified Acute Physiology Score; SOFA = Sequential Organ-Failure Assessment; SRD = systemic rheumatic diseases; VA-ECMO = venoarterial-extracorporeal membrane oxygenation.

The multiple Cox proportional hazards model used backward-stepwise variable elimination (with the variable exit threshold set at *P*>0.10). All potential explanatory variables included in the multivariable analyses were subjected to colinearity analysis with a correlation matrix. Variables associated with one another were not included in the model. Statistical significance was defined as *P*<0.05.

| **e-Table E5.** In-ICU Characteristics and Outcomes of the 42 VV-ECMO–Treated SRD Patients: Hospital Survivors vs. Nonsurvivors | | | | |
| --- | --- | --- | --- | --- |
| **Characteristic** | ***n*/*n**** | **Survivors**  ***n*=21** | **Nonsurvivors**  ***n*=21** | ***P***  **Value** |
| Female |  | 12 (57.1) | 14 (66.7) | 0.5 |
| Age at admission, years |  | 38.6±17.1 | 47.8±15.1 | 0.07 |
| Symptom-onset-to-hospital interval, days |  | 5 [0–12] | 9 [0–29] | 0.4 |
| Symptom-onset-to-ICU interval, days |  | 11 [4–23] | 28 [12–47] | 0.01 |
| Hospital-to-ICU interval, days |  | 5 [2–9] | 15 [6–32] | 0.001 |
| Days in ICU |  | 26 [16–69] | 20 [7–39] | 0.08 |
| Days in hospital |  | 70 [38–111] | 39 [26–58] | 0.019 |
| Pre-admission SRD |  |  |  |  |
| Connective tissue diseases |  | 9 (42.9) | 12 (57.1) | 0.4 |
| Vasculitides |  | 7 (33.3) | 2 (9.5) | 0.06 |
| Corticosteroids |  | 8 (38.1) | 13 (61.9) | 0.1 |
| Immunosuppressant(s) |  | 6 (28.6) | 11 (52.4) | 0.1 |
| Lung involvement |  | 16 (76.2) | 15 (71.4) | 0.7 |
| Heart involvement |  | 1 (4.8) | 2 (9.5) | 0.5 |
| In-ICU SRD |  |  |  |  |
| Flare-related admission |  | 14 (66.7) | 17 (81) | 0.3 |
| In-ICU diagnosis |  | 11 (52.4) | 8 (38.1) | 0.4 |
| Corticosteroids |  | 16 (76.2) | 16 (76.2) | 0.9 |
| Immunosuppressant |  | 9 (42.9) | 9 (42.9) | 0.9 |
| Reason for ICU admission |  |  |  |  |
| Respiratory failure |  | 21 (100) | 21 (100) | n/a |
| Organ failure at ICU admission |  |  |  |  |
| Day-0 SAPS II |  | 59 [54–67] | 63 [50–75] | 0.8 |
| Day-0 SOFA score |  | 12 [9–14] | 14 [10–18] | 0.2 |
| VV-ECMO |  |  |  |  |
| Pre-ECMO PaO_2_/FiO_2_ ratio | 17/12 | 55 [45–70] | 54 [50–77] | 0.6 |
| RESP score | 20/21 | 2 [–0.5 to 3] | –2 [–4 to 0] | 0.001 |
| Days on ECMO |  | 14 [7–66] | 11 [8–27] | 0.8 |
| ECMO complication |  |  |  |  |
| Limb ischemia |  | 1 (4.8) | 1 (4.8) | 0.9 |
| Insertion-site hemorrhage |  | 2 (9.5) | 6 (28.6) | 0.1 |
| Cannula-related infection |  | 4 (19.0) | 5 (23.8) | 0.7 |
| In-ICU organ support |  |  |  |  |
| Dobutamine |  | 2 (9.5) | 2 (9.5) | 0.9 |
| Vasopressors |  | 18 (85.7) | 20 (95.2) | 0.3 |
| Mechanical ventilation |  | 21 (100) | 20 (95.2) | 0.3 |
| Renal replacement therapy |  | 10 (47.6) | 15 (71.4) | 0.1 |
| Highest in-ICU value |  |  |  |  |
| Arterial lactate, mmol/L |  | 3.6 [2.6–8.1] | 10 [4.3–15.2] | 0.02 |
| Troponin, ULN | 17/20 | 5.1 [1.5–8] | 3.8 [0.5–11.6] | 0.5 |
| Serum creatinine, µmol/L | 21/18 | 104 [79–362] | 123 [67–178] | 0.4 |
| Lowest in-ICU value |  |  |  |  |
| Platelet count, G/L |  | 78 [34–118] | 28 [9–46] | 0.003 |
| Prothrombin time, % |  | 50 [39–59] | 38 [17–57] | 0.08 |
| Outcome |  |  |  |  |
| In-ICU–acquired infection |  | 12 (57.1) | 18 (85.7) | 0.04 |
| Ventilator-associated pneumonia |  | 9 (42.9) | 17 (81) | 0.01 |
| Fungal infection |  | 2 (9.5) | 7 (33.3) | 0.06 |
| Lung transplantation |  | 2 (9.5) | 0 (0) | 0.1 |
| Weaning |  | 19 (90.5) | 2 (9.5) | <0.0001 |
| In-ICU mortality |  | 0 (0) | 20 (95.2) | <0.0001 |
| Abbreviations: ICU = intensive care unit; LVAD = left ventricular assist device; LVEF = left ventricle ejection fraction; PaO_2_/FiO_2_ = partial pressure of arterial oxygen/fraction of inspired oxygen; RESP = Respiratory Extracorporeal Membrane Oxygenation Survival Prediction; SAPS = Simplified Acute Physiology Score; SOFA = Sequential Organ-Failure Assessment; SRD = systemic rheumatic disease; ULN = upper limit of normal value; VV-ECMO = venovenous-extracorporeal membrane oxygenation; VTI = velocity-time integral.  Continuous variables are expressed as mean±standard deviation or median [interquartile range] and compared with Student’s *t*-test or Wilcoxon’s rank test; categorical variables are expressed as No. (%) and compared with χ^2^ tests.  *Numbers of survivor/nonsurvivor data available. | | | | |

| **eTable E6.** Univariable and Multivariable Analyses of Factors Associated with In-Hospital Mortality for the 42 VV-ECMO–Treated SRD Patients | | | | | | | |  |
| --- | --- | --- | --- | --- | --- | --- | --- | --- |
|  | **Univariable Analysis** | | |  | **Multivariable Analysis** | | | |
| **Factor** | **HR** | **95% CI** | ***P* Value** |  | **HR** | **95% CI** | ***P* Value** | |
| Age ≥40 yr | 1.5 | 0.6–3.7 | 0.4 |  |  |  |  | |
| Women | 1.2 | 0.5–3.0 | 0.7 |  |  |  |  | |
| Pre-ICU–admission SRD lung involvement | 0.6 | 0.2–1.5 | 0.2 |  |  |  |  | |
| Pre-ICU–admission SRD heart involvement | 1.7 | 0.4–7.3 | 0.5 |  |  |  |  | |
| Vasculitis | 0.3 | 0.07–1.2 | 0.09 |  | **0.1** | **0.02–0.8** | **0.02** | |
| Corticosteroids before admission | 1.9 | 0.8–4.5 | 0.1 |  |  |  |  | |
| Immunosuppressant(s) before admission | 2.0 | 0.9–4.8 | 0.1 |  |  |  |  | |
| In-ICU SRD diagnosis | 0.6 | 0.2–1.4 | 0.3 |  |  |  |  | |
| Day-0 SAPS II ≥70 | 2.3 | 0.9–5.8 | 0.07 |  | **8.8** | **2.1–37.6** | **0.003** | |
| Day-0 SOFA score ≥16 | 2.9 | 1.2–7.0 | 0.02 |  |  |  |  | |
| RESP score ≤ 0 | 4.5 | 1.5–13.6 | 0.007 |  |  |  |  | |
| Flare-related admission | 1.8 | 0.6–5.5 | 0.3 |  |  |  |  | |
| Corticosteroids in the ICU | 0.9 | 0.3–2.4 | 0.8 |  |  |  |  | |
| Immunosuppressant(s) in the ICU | 0.9 | 0.4–2.2 | 0.8 |  |  |  |  | |
| Vasopressors | 2.4 | 0.3–18.1 | 0.4 |  |  |  |  | |
| Mechanical ventilation | 0.1 | 0.02–1.3 | 0.09 |  |  |  |  | |
| Renal replacement therapy | 2.4 | 0.9–6.2 | 0.07 |  |  |  |  | |
| ICU-acquired infection | 2.7 | 0.8–9.1 | 0.1 |  |  |  |  | |
| ICU-acquired VAP | 3.2 | 1.1–9.4 | 0.04 |  | **15.8** | **3.2–77.8** | **0.001** | |
| Highest in-ICU value |  |  |  |  |  |  |  | |
| Arterial lactate ≥7.5 mmol/L | 3.3 | 1.4–8.1 | 0.007 |  | **3.0** | **1.1–8.2** | **0.03** | |
| Bilirubin ≥125 µmol/L | 4.0 | 1.6–9.8 | 0.002 |  |  |  |  | |
| Lowest in-ICU value |  |  |  |  |  |  |  | |
| Platelet count <50 G/L | 5.2 | 1.7–15.6 | 0.003 |  |  |  |  | |

*Definition of abbreviations:* ICU = intensive care unit; RESP = Respiratory Extracorporeal Membrane Oxygenation Survival Prediction; SAPS = Simplified Acute Physiology Score; SOFA = Sequential Organ-Failure Assessment; SRD = systemic rheumatic diseases; VV-ECMO = venovenous-extracorporeal membrane oxygenation; VAP = ventilator-associated pneumonia.

The multiple Cox proportional hazards model used backward-stepwise variable elimination (with the variable exit threshold set at *P*>0.10). All potential explanatory variables included in the multivariable analyses were subjected to colinearity analysis with a correlation matrix. Variables associated with one another were not included in the model. Statistical significance was defined as *P*<0.05.
